# Converging Pathways in Cancer Biology: How Do the Microbiome, Angiogenesis, Senescence, Fibroblast Plasticity, and Immunotherapy Intertwine?

**DOI:** 10.3390/cancers18050826

**Published:** 2026-03-04

**Authors:** Inamul Haque, Suman Kambhampati, Sushanta K. Banerjee

**Affiliations:** 1 Cancer Research Unit, Kansas City Veterans Affairs Medical Center, Kansas City, MO 64128, USA; suman.kambhampati@va.gov (S.K.); sushanta.banerjee@va.gov (S.K.B.); 2 Department of Cancer Biology, University of Kansas Medical Center, Kansas City, KS 66160, USA; 3 Department of Math, Science and Business Technology, Kansas City Kansas Community College, Kansas City, KS 66112, USA; 4 Department of Pathology and Laboratory Medicine, University of Kansas Medical Center, Kansas City, KS 66160, USA

## 1. Introduction

Cancer continues to be a major cause of death, with an anticipated 2,114,850 new cases and almost 626,140 deaths from the disease in 2026 [1]. It is increasingly being recognized as a systemic disease driven not only by intrinsic genetic alterations but also by the dynamic interplay between malignant cells and their surrounding TME. The TME is a complex and dynamic ecosystem of cellular and non-cellular components where cancer grows. The cellular components include cancer cells, infiltrating immune cells (T lymphocytes, B lymphocytes, dendritic cells, natural killer cells, macrophages, neutrophils, and myeloid-derived suppressor cells), stromal cells (CAFs, adipocytes, endothelial cells, blood cells, and neuroendocrine cells), and non-cellular components comprise signaling molecules and proteins of the extracellular matrix (ECM) such as collagen, elastin, fibronectin, laminin, and tanascin [2]. The TME is a crucial factor in cancer development, progression, and metastasis due to the complex interplay among its components. Cancer cells influence the microenvironment by releasing extracellular signals, promoting tumor angiogenesis and inducing immune tolerance, whereas stromal and immune cells help cancer cells evade immune recognition, provide a pro-tumorigenic environment, develop drug resistance, metastasize, and exhibit increasingly malignant behaviors [3]. The ECM serves to internally structuralize its cellular constituents and promotes feedback through biochemical and biomechanical actions executed by a complex of fibrous proteins, glycoproteins, growth factors, and glycosaminoglycans [4]. The properties of the ECM support the integrity of the local microenvironment by maintaining mechanical function and structure, regulating growth factor and nutrient supply and concentration, and establishing adhesion gradients [5]. During tumor progression, ECM homeostasis and the dysregulation of fibrous proteins initiate the metastasis of tumor cells via the circulatory and lymphatic pathways of the TME to other organ tissues [6]. The identification of microbial communities, including *Helicobacter pylori* (*H. pylori*), *Porphyromonas gingivalis* (*P. gingivalis*), and *Fusobacterium nucleatum* (*F. nucleatum*), in gastric, oral, and colorectal cancer, respectively, has turned the spotlight onto the diverse roles of the microbiota in carcinogenesis, progression, and therapeutic response [7,8,9]. These elements do not act in isolation; rather, they form a complex signaling ecosystem where pathways such as Wnt/β-catenin, TGF-β/SMAD, NF-κB, and Hedgehog signaling intersect with metabolic and inflammatory cues to influence proliferation, invasion, angiogenesis, immune evasion, and therapeutic efficacy.

Recent evidence underscores the fact that microenvironmental factors can amplify or attenuate the efficacy of systemic therapies, including chemotherapy, targeted agents, and immunotherapy. For example, desmoplastic stroma and aberrant vasculature limit the efficacy of therapeutic drugs by blocking their delivery to tumor cells [10], while the senescence-associated secretory phenotype ( SASP) can promote resistance and metastasis [11]. Similarly, intratumoral microbiota can either suppress immune surveillance or enhance antigenicity, thereby modulating responses to checkpoint inhibitors and cellular immunotherapies [12]. These insights highlight the urgent need for therapeutic strategies that move beyond single-target inhibition toward integrated approaches addressing multiple microenvironmental axes.

The five papers featured in this Special Issue collectively shed light on these dimensions of tumor biology. They explore: (i) the gastric and intratumoral microbiomes as modulators of carcinogenesis and immunotherapy outcomes; (ii) the vascular niche and 20 years of clinical experience with anti-VEGF therapy; (iii) SASP-driven signaling in head and neck cancers and its therapeutic implications; (iv) adipose stromal cell-derived CAFs orchestrating ECM remodeling and metastatic progression in pancreatic ductal adenocarcinoma; (v) the interface between microbial ecology and cellular immunotherapy. Together, these contributions provide actionable insights for clinical practice—spanning early detection, patient stratification, therapy sequencing, and combination design—while charting a roadmap for next-generation trials that integrate microbiome modulation, stromal targeting, vascular normalization, and immune activation. The featured studies collectively underscore a shift toward multi-axis therapeutic designs. To provide a clear overview of the clinical and molecular scope of these contributions, Table 1 summarizes the core focus, key molecular targets, and clinical indications for each of the five papers.

## 2. Microbiome–Tumor Crosstalk: From Gastric Ecology to Intratumoral Niches

Al Matouq et al. provided a rapidly expanding body of evidence demonstrating the intricate relationship between the human microbiome and gastric cancer (GC), emphasizing how microbial dysbiosis influences tumor development through genetic instability, chronic inflammation, immune modulation, and metabolic shifts [13]. While *H. pylori* remains a primary driver of GC, the article highlights that other stomach- and oral-derived bacteria also play significant roles in creating pro-tumorigenic environments by producing genotoxins and carcinogenic metabolites, such as N-nitroso compounds. In this review, the authors emphasize the potential of microbiome signatures as biomarkers for early GC detection and discusses therapeutic strategies, including probiotics, prebiotics, synbiotics, fecal microbiota transplantation, engineered microbes, and nanotechnology. Additionally, it examines how the microbiota impacts chemotherapy efficacy, toxicity, and immunotherapy response and proposes microbiome modulation as a promising adjunct to conventional cancer treatments. Despite advances in this area, challenges such as individual variability and safety concerns necessitate further research to develop personalized, microbiome-based interventions for GC management.

The review by Leonov et al. examines the emerging role of the intratumoral microbiome, comprising bacteria, fungi, and viruses, in cancer progression and its impact on cellular immunotherapy [14]. It highlights the multifaceted effects on mechanisms and signaling pathways, such as WNT/β-catenin, NF-κB, and PI3K, that influence tumor initiation and progression. The authors also discuss how some microbiome communities exert antitumor immunity by inhibiting the activation of pathways such as HIF-1α/NFkB, TLR4, RhoA/ROCK, and MEK/ERK through the production of immunomodulatory metabolites. The intratumoral microbiome shapes the tumor immune microenvironment, thereby affecting responses to therapies such as CAR-T cells, TILs, and checkpoint inhibitors. Evidence suggests that microbial diversity and composition correlate with treatment outcomes, and innovative strategies—such as engineered bacteria, probiotics, and microbiome-targeted adjuncts—are being explored to improve immunotherapy efficacy. The authors call for mechanistic studies, multi-omics approaches, and clinical trials to validate microbial biomarkers and develop personalized microbiome-based interventions for cancer treatment.

## 3. Angiogenesis: Lessons from Bevacizumab and Beyond

Chitoran et al. provide a rigorous and comprehensive analysis of tumor angiogenesis and the VEGF/VEGFR signaling axis and catalog the clinical arc of bevacizumab across colorectal, lung, ovarian, cervical, brain, glioblastoma, and renal cancers [15]. The review underscores consistent progression-free survival gains across several indications, variable overall survival benefits, and recognized safety signals (e.g., hemorrhage and hypertension). Despite the benefits detailed in the review, the authors address numerous controversies, such as the methodological errors of clinical trials, FDA withdrawal for breast cancer patients, severe adverse effects, and resistance mechanisms that limit long-term efficacy. This paper contextualizes newer anti-angiogenic strategies, resistance mechanisms (e.g., compensatory pathways and hypoxia-selected clones), and combinatorial regimens with tyrosine kinase inhibitors (TKIs) and immunotherapy (e.g., atezolizumab + bevacizumab + chemo in non-small-cell lung cancer) that aim to normalize vasculature and reset immune trafficking.

## 4. Senescence-Associated Secretory Phenotype (SASP) in Head and Neck Cancers

Alam and colleagues explore the dual nature of SASP in head and neck cancers (HNCs), emphasizing its paradoxical impact on tumor biology and therapy [16]. While the SASP initially supports tumor suppression via immunosurveillance and growth arrest, its prolonged activity fosters a pro-inflammatory and pro-angiogenic microenvironment that promotes EMT, ECM remodeling, cancer stemness, and radio/therapy resistance. In their review, the authors connect persistent DNA damage response (DDR) signaling to NF κB/STAT3-driven cytokine networks (IL-6/IL-8), highlight SASP’s roles in radio- and chemoresistance, and investigate senolytic/senomorphic strategies (navitoclax and JAK/mTOR inhibitors) and IL-6/IL-8 blockade, which aim to attenuate resistance while preserving beneficial senescence cues. They also emphasize miRNA circuits (e.g., miR-146a, miR-34a, miR-21, and miR-503) that gate SASP intensity and EMT programs, proposing biomarker-guided personalization of senescence modulation. The review underscores the need for biomarker discovery, personalized SASP-targeted therapies, and the integration of advanced models, such as organoids and single-cell analysis, to overcome treatment resistance and improve outcomes in HNCs.

## 5. Adipose-Derived CAFs: SFRP4/LINC01614 Orchestrate Wnt–TGFβ–ECM

Cancer-associated fibroblasts are the main component of the TME and can support the growth and invasion of cancer cells through multiple mechanisms [18]. CAFs in the TME are associated with worse prognosis, drug resistance, and disease relapse in multiple solid cancers [19]. Emerging evidence suggests that CAFs originate from normal resident tissue fibroblasts, bone marrow-derived mesenchymal stem cells, tumor epithelial cells (via EMT), endothelial cells (via endothelial-to-mesenchymal transition (EndMT)), pericytes (via pericyte-to-fibroblast transition (PFT)) and stellate cells (mainly seen in desmoplastic reactions in pancreatitis and pancreatic cancer) [20]. Moreover, CAFs contribute to immune evasion of cancer cells and to an immunosuppressive milieu by upregulating cytokine production and immune checkpoint ligands, inhibiting CD8^+^ antitumor functions or polarizing T-cell subsets toward Th2, thereby favoring the recruitment and differentiation of Tregs. The remodeling of ECM components, including collagen, fibronectin, and matrix metalloproteinases (MMPs), by CAFs reduces the infiltration of effector T cells. It has been shown that CAFs recruit pro-tumorigenic M2 macrophages, Treg cells, and MDSCs into the TME [21]. Secreted frizzled-related protein 4 (SFRP4), a Wnt signaling modulator, has been found to act as either a tumor suppressor or a pro-oncogenic factor, depending on the tissue type and TME. In breast cancer, in a tumor-suppressive role, SFRP4+ CAFs secrete SFRP4 to inhibit Wnt signaling in cancer cells, thereby further blocking cell migration and EMT. In contrast, Yang et al. demonstrated that increased expression of SFRP4 in PanINs and PDAC tissues correlates with FOXP3+ Treg cell infiltration, suggesting its role in shaping an immunosuppressive TME and in poor survival in PDAC patients [22]. The preclinical experimental study by Kolonin and coworkers examines how SFPR4 is involved in the transition of adipose stromal cells (ASCs) into cancer-associated fibroblasts (CAFs) and in the promotion of PDAC progression [17]. Using human ASC–PDAC co-culture and mouse models, the study reveals that PDAC cells activate Wnt, TGFβ, and Hedgehog signaling in ASCs, driving ECM remodeling and desmoplasia. The authors discovered that two key regulators, LINC01614 (a long non-coding RNA) and SFRP4, are essential for this fibroblastic transition. The knockout of either gene in ASCs suppresses Wnt/TGFβ signaling, ECM gene expression (including COL11A1), and PDAC cell migration, while SFRP4 loss also reduces invasion and metastasis in vivo. Mechanistically, ASC-derived SFRP4 and LINC01614 form a feedback loop that enhances cancer cell aggressiveness by activating Wnt/TGFβ and Hedgehog signaling. Importantly, SFRP4 knockout in mice diminishes tumor growth, desmoplasia, and liver micro-metastases, positioning SFRP4 as a promising therapeutic target to disrupt stromal–tumor crosstalk and limit PDAC progression. Recently, we discovered that CCN1 expression in PDAC cells can transform normal fibroblasts into CAFs by inducing TGF-β and α-smooth muscle actin (α-SMA), thereby driving desmoplasia, EMT, tumor progression, and metastasis (unpublished data).

## 6. Future Directions for CAF–Microbiome–Immunotherapy Triads

As discussed in the previous section, CAFs are recognized as a central actor in the TME architecture, influencing tumor growth, ECM remodeling, EMT, angiogenesis, metastasis, and therapeutic resistance, thereby contributing to poor treatment outcomes for cancer patients. Therefore, there is an urgent need to develop novel therapies aimed at targeting CAFs. Several strategies have been adopted in this regard. One strategy is to inhibit CAF-related signaling pathways, such as TGFβ, Sonic hedgehog (SHh), connective tissue growth factor (CTGF), and CXCL12. A preclinical study by Pang and colleagues explored a two-stage combination therapy approach using the TGF-β receptor I inhibitor SB525334 (SB) and docetaxel-loaded micelles (DTX-M) [23]. They found that SB reduces myofibroblast (myCAF) populations and ECM stiffness, thereby improving DTX-M delivery to the tumor and inducing apoptosis in both tumor cells and CAFs. Pamrevlumab, a human monoclonal antibody that antagonizes CTGF, significantly inhibited the progression of pancreatic cancer, mesothelioma, and melanoma in an orthotopic nude mouse model [24,25,26,27]. It has also been discovered that CCN1-induced activation of SHh signaling might be necessary for PDAC cell migration and the tumorigenicity of CCN1-enriched pancreatic cancer cells in a xenograft model in nude mice [28]. In another study, inhibiting SHh signaling with LDE225 (Sonidegib) enhanced blood vessel normalization, ECM reduction, and gemcitabine penetration to the tumor site [29]. Moreover, CXCL12-mediated conversion of normal fibroblasts to CAFs is significantly suppressed by CXCL12 antagonist AMD3100 in an orthotopic mouse model [30]. Although this strategy shows promise in preclinical studies, its clinical efficacy in patients remains disappointing [31].

Another strategy aims to revert CAFs to a quiescent state. In this context, Minnelide, vitamin D, and vitamin A effectively reprogram activated CAFs to a quiescent non-proliferative state and promote drug delivery to the pancreatic tumor in a mouse model [32,33,34]. Freag et al. tested the efficacy of engineered exosome biogenesis inhibitor-loaded nanoliposomes combined with anti-αPD-L1 antibodies in lung cancer syngeneic mice and found that these nanoparticles not only prevent the generation of new CAFs but also revert pre-existing CAFs to a quiescent state, thereby enhancing the efficacy of anti-αPD-L1 antibodies in their augmentation of the antitumor immune response [35]. All-Trans Retinoic Acid (ATRA), as a single agent or in combination with gemcitabine-nab-paclitaxel or pemprolizumab, led to enhanced antitumor effects by suppressing ECM remodeling and inhibiting tumor cell invasion [36]. While these strategies have shown encouraging results, clinical trials are still in the early stages.

Given that fibroblast activation protein (FAP) is selectively overexpressed in CAFs but minimally expressed in quiescent fibroblasts, it has emerged as a high-value therapeutic target. Current strategies, including FAP inhibitors (FAPIs), FAP-directed chimeric antigen receptor T-cell (CAR-T) therapy, and FAP-targeting vaccines, have demonstrated significant efficacy in preclinical models by modulating the TME to inhibit progression, reduce vascularization, and potentiate immune-mediated tumor clearance. Although several early FAPIs designed by Jansen et al. and researchers at the University of Heidelberg demonstrated significant inhibition of the endopeptidase activity of FAP, they had limited tumor retention time [37,38]. Currently, FAPI variants labeled with therapeutic radionuclides, such as ^90^Y-FAPI-46, ^68^Ga-FAPI-46, ^18^F-FAPI-74, ^177^LU-FAPI-2286, and ^99m^Tc-FAPI-34, display longer retention times and better responses and have been evaluated in preclinical [38,39,40,41] and clinical studies (NCT05262855, NCT05641896, and NCT04939610). CAR-T therapy has shown satisfactory results by using dual-function CAR-T cells that target both mesothelin-expressing tumor cells and FAP-positive CAFs, thereby exhibiting superior antitumor efficacy in patient-derived and mouse pancreatic cancer models [42]. Vaccines directed against FAP exploit host immunity to eradicate FAP^+^ CAFs. Preclinical investigations demonstrate that an adenoviral vector encoding FAP induces robust CD8^+^ T-cell activation, resulting in significant suppression of both primary and metastatic disease in multidrug-resistant mouse models [43]. Results from Chen et al. indicated that an FAP-modified whole-cell tumor vaccine enhanced the infiltration of CD8^+^ T lymphocytes and suppressed the accumulation of immunosuppressive cells in the TME [44].

Future research on CAF-targeted therapies will benefit from a more refined understanding of CAF heterogeneity, supported by advances in single-cell transcriptomics, secretome profiling, and high-resolution imaging capable of tracking dynamic FAP expression within the tumor microenvironment. Continued improvement of CAF classification systems will help delineate functionally distinct subsets and clarify their contributions to tumor progression, immune modulation, and therapeutic resistance. These insights are expected to guide the development of more selective and effective strategies for targeting FAP-positive CAFs. Combining CAF-directed interventions with immunotherapies, chemotherapies, or targeted agents may enhance intratumoral drug delivery, alleviate immune exclusion, and reduce resistance mechanisms. Ultimately, integrating CAF-focused approaches into personalized treatment regimens could support the development of more innovative therapeutic modalities and improve clinical outcomes.

Preclinical and early clinical studies suggest that intratumoral microbiomes can impact tumor progression and the host’s immune response, thereby significantly shaping immunosurveillance and affecting the efficacy and safety of cancer immunotherapy [45,46,47,48]. Intratumoral microbes such as *Sphingobacterium multivorum* and *Bacteroides intestinalis* orchestrate immunosuppressive chemokine networks (CCL20 and CXCL8), promoting the accumulation of regulatory T cells (Tregs) and myeloid-derived suppressor cells (MDSCs) and inhibiting CD8^+^ T-cell activity in the TME, thereby undermining immune checkpoint inhibitor (ICI) responses [49]. Moreover, the total intratumoral microbial burden, independent of specific taxa, correlates with immunosuppressive phenotypes characterized by neutrophil-rich, T-cell-depleted microenvironments and resistance to ICIs [50]. Mechanistically, bacterial colonization activates PRDX1-driven glycolysis in hepatocellular carcinoma, increasing lactate production, which suppresses NK and CD8^+^ T-cell function and upregulates exhaustion markers TIM-3 and LAG-3, thereby reducing the efficacy of PD-1 inhibitors [51]. Certain metabolites produced by microbes, such as butyrate, a short-chain fatty acid (SCFA), effectively inhibit the progression of melanoma lung metastases by attracting T helper (Th) cells to the TME via upregulation of the CCL20 chemokine [52]. Furthermore, butyrate produced by *Roseburia intestinalis* and/or *Akkermansia municiphilia* may improve the efficacy of ICIs (anti-PD1/PD-L1/CTLA-4) by promoting CD8^+^ activity in an orthotopic melanoma mouse model and melanoma patients [53]. In addition, oral supplementation of a probiotic cocktail containing live *Bifidobacterium* spp. inhibited melanoma tumor growth by improving anti-PD-L1 efficacy through the enhancement of CD8^+^ activity [54]. Clinically, fecal microbiota transplantation (FMT) from anti-PD-1 responders to non-responders has successfully converted a subset of patients with melanoma and other solid tumors into responders, demonstrating immunotherapy re-sensitization [55]. Moreover, results from a multicenter phase I trial (NCT03772899) show that combining FMT with PD-1 inhibitors nivolumab and pembrolizumab in patients with advanced melanoma modulates the antitumor immune response and enhances anti-PD-1 efficacy [56]. The findings from the FMT-LUMINate trial, a phase II study (NCT04951583), suggest that FMT plus dual PD-1/CTLA-4 blockade in non-small-cell lung cancer and melanoma patients effectively sensitizes tumors by eliminating immunosuppressive microbes and expanding circulating CD8^+^ memory T cells [57].

These findings position the tumor microbiome as a dynamic determinant of immune response, highlighting strategies such as microbiome modulation and bacterial metabolism targeting to enhance cancer immunotherapy, paving the way for more effective and personalized cancer treatments.

## 7. Conclusions and Perspectives

This Special Issue emphasizes a unifying insight: cancer control requires orchestration of the microenvironment. Gastric and intratumoral microbiomes can either amplify oncogenic inflammation or expand antigenic breadth; the vascular niche gates delivery and immunity; senescence networks determine whether DDR fuels clearance or chronic resistance; and adipose-derived CAFs remodel the ECM to either barricade immunity or, when reprogrammed, re-open access. The translational mandate is clear: design multimodal, biomarker-driven combinations that time and tune interventions across these axes and evaluate them in trials with harmonized microbiome and microenvironment endpoints. If we do, we can move beyond incremental gains toward durable, system-level control of cancer.

## Figures and Tables

**Table 1:** Molecular targets and clinical significance of the featured contributions.

Core Focus	Key Molecular Targets	Clinical/Therapeutic Indications	Study
Gastric Microbiome & Pathogenesis	Virulence factors (VacA & CagA); Wnt/NF-κB pathways; TLR activation (LPS).	Early detection biomarkers; fecal microbiota transplantation (FMT); precision probiotics for gastric cancer prevention.	Al Matouq et al. [13]
Intratumoral Microbiota & Immunotherapy	TIGIT/CEACAM1; WNT-β-catenin & PI3K/AKT signaling; HLA-presented microbial peptides.	Optimization of CAR-T-cell efficacy; “living therapeutics” (engineered microbes); predictive biomarkers for ICI response.	Leonov et al. [14]
Anti-Angiogenic Therapy (20 yr Review)	VEGF-A; VEGFR-1/2; VE-cadherin phosphorylation; PI3K/AKT & RAS/MAPK axes.	Ovarian cancer maintenance; mCRC; NSCLC; vascular normalization to enhance chemotherapy delivery.	Chitoran et al. [15]
SASP in Head & Neck Cancers (HNCs)	IL-6, IL-8, & TGF-β; DDR signaling; p16INK4a/p21; NF-κB & mTOR signaling.	Radiosensitization; use of senomorphics (mTOR/JAK inhibitors) and senolytics to overcome treatment resistance.	Alam et al. [16]
CAF Plasticity & Desmoplasia	SFRP4; LINC01614; TGF-β & Wnt ligands; PKA-mediated phosphorylation.	“Migrastatic” therapy for PDAC; reduction in desmoplasia to improve drug penetration and prevent metastasis.	Rupert et al. [17]
